# Childhood and adolescence factors and multiple sclerosis: results from the German National Cohort (NAKO)

**DOI:** 10.1186/s12883-024-03620-4

**Published:** 2024-04-13

**Authors:** Anja Holz, Nadia Obi, Wolfgang Ahrens, Klaus Berger, Barbara Bohn, Hermann Brenner, Beate Fischer, Julia Fricke, Amand Führer, Sylvia Gastell, Karin Halina Greiser, Volker Harth, Jana-Kristin Heise, Bernd Holleczek, Thomas Keil, Carolina J. Klett-Tammen, Michael Leitzmann, Wolfgang Lieb, Claudia Meinke-Franze, Karin B. Michels, Rafael Mikolajczyk, Katharina Nimptsch, Annette Peters, Tobias Pischon, Oliver Riedel, Tamara Schikowski, Sabine Schipf, Börge Schmidt, Matthias B. Schulze, Andreas Stang, Kerstin Hellwig, Karin Riemann-Lorenz, Christoph Heesen, Heiko Becher

**Affiliations:** 1 Institute of Medical Biometry and Epidemiology (IMBE), University Medical Center Hamburg-Eppendorf (UKE), Martinistr. 52, 20246 Hamburg, Germany; 2 Institute of Tumor Biology, University Medical Center Hamburg-Eppendorf (UKE), Hamburg, Germany; 3 Institute for Occupational and Maritime Medicine (ZfAM), University Medical Center Hamburg-Eppendorf (UKE), Hamburg, Germany; 4 Leibniz Institute for Prevention Research and Epidemiology – BIPS, Bremen, Germany; 5 University Bremen, Bremen, Germany; 6 Institute of Epidemiology and Social Medicine, University of Münster, Münster, Germany; 7 NAKO e.V., Heidelberg, Germany; 8 Division of Clinical Epidemiology and Aging Research, German Cancer Research Center (DKFZ), Division of Preventive Oncology, German Cancer Research Center (DKFZ), Heidelberg, Germany; 9 Institute for Epidemiology and Preventive Medicine, University of Regensburg, Regensburg, Germany; 10 Institute of Social Medicine, Epidemiology and Health Economics, Charité – Universitätsmedizin Berlin, Berlin, Germany; 11 Institute for Medical Epidemiology, Biometrics and Informatics (IMEBI), Interdisciplinary Center for Health Sciences, Medical School of the Martin Luther University Halle-Wittenberg, Halle (Saale), Germany; 12 NAKO Study Center, German Institute of Human Nutrition Potsdam-Rehbruecke, Nuthetal, Germany; 13 Division of Cancer Epidemiology, German Cancer Research Center (DKFZ), Heidelberg, Germany; 14 Helmholtz Centre for Infection Research (HZI), Hannover, Germany; 15 Saarland Cancer Registry, Saarbrücken, Germany; 16 Institute of Clinical Epidemiology and Biometry, University of Würzburg, Würzburg, Germany; 17 State Institute of Health I, Bavarian Health and Food Safety Authority, Erlangen, Germany; 18 Department for Epidemiology, Helmholtz Centre for Infection Research (HZI), Brunswick, Germany; 19 Institute of Epidemiology, Kiel University, Kiel, Germany; 20 Institute for Community Medicine, University Medicine Greifswald, Greifswald, Germany; 21 Institute for Prevention and Cancer Epidemiology, Faculty of Medicine and Medical Center, University of Freiburg, Freiburg, Germany; 22 Max-Delbrück-Center for Molecular Medicine in the Helmholtz Association (MDC), Molecular Epidemiology Research Group, Berlin, Germany; 23 Institute of Epidemiology, Helmholtz Zentrum München - German Research Center for Environmental Health (GmbH), Neuherberg, Germany; 24 Chair of Epidemiology, Institute for Medical Information Processing, Biometry and Epidemiology, Ludwig-Maximilians-Universität München, Munich, Germany; 25 Max-Delbrück-Center for Molecular Medicine in the Helmholtz Association (MDC), Biobank Technology Platform, Berlin, Germany; 26 Charité - Universitätsmedizin Berlin, corporate member of Freie Universität Berlin and Humboldt-Universität zu Berlin, Berlin, Germany; 27 IUF - Leibniz Research Institute for Environmental Medicine, Düsseldorf, Germany; 28 Institute for Medical Informatics, Biometry and Epidemiology, Medical Faculty, University Duisburg-Essen, Essen, Germany; 29 Department of Molecular Epidemiology, German Institute of Human Nutrition Potsdam-Rehbruecke, Nuthetal, Germany; 30 Institute of Nutritional Science, University of Potsdam, Nuthetal, Germany; 31 School of Public Health, Department of Epidemiology Boston University, 715 Albany Street, Talbot Building, Boston, MA 02118 USA; 32 Katholisches Klinikum Bochum, Neurology Clinic, Clinic of Ruhr-Universität Bochum, Bochum, Germany; 33 Center for Molecular Neurobiology Hamburg (ZMNH), Institute of Neuroimmunology and Multiple Sclerosis (INIMS), University Medical Center Hamburg-Eppendorf (UKE), Hamburg, Germany; 34 Department of Neurology, University Medical Center Hamburg-Eppendorf (UKE), Hamburg, Germany; 35 Institute of Global Health, University Hospital Heidelberg, Heidelberg, Germany

**Keywords:** Multiple Sclerosis, Childhood, Adolescence, Epidemiology

## Abstract

**Background:**

Multiple Sclerosis (MS) represents the most common inflammatory neurological disease causing disability in early adulthood. Childhood and adolescence factors might be of relevance in the development of MS. We aimed to investigate the association between various factors (e.g., prematurity, breastfeeding, daycare attendance, weight history) and MS risk.

**Methods:**

Data from the baseline assessment of the German National Cohort (NAKO) were used to calculate adjusted hazard ratios (HR) and 95% confidence intervals (CI) for the association between childhood and adolescence factors and risk of MS. Analyses stratified by sex were conducted.

**Results:**

Among a total of 204,273 participants, 858 reported an MS diagnosis. Male sex was associated with a decreased MS risk (HR 0.48; 95% CI 0.41–0.56), while overweight (HR 2.03; 95% CI 1.41–2.94) and obesity (HR 1.89; 95% CI 1.02–3.48) at 18 years of age compared to normal weight were associated with increased MS risk. Having been breastfed for ≤ 4 months was associated with a decreased MS risk in men (HR 0.59; 95% CI 0.40–0.86) compared to no breastfeeding. No association with MS risk was observed for the remaining factors.

**Conclusions:**

Apart from overweight and obesity at the age of 18 years, we did not observe considerable associations with MS risk. The proportion of cases that can be explained by childhood and adolescence factors examined in this study was low. Further investigations of the association between the onset of overweight and obesity in childhood and adolescence and its interaction with physical activity and MS risk seem worthwhile.

**Supplementary Information:**

The online version contains supplementary material available at 10.1186/s12883-024-03620-4.

## Background

Multiple Sclerosis (MS) is an inflammatory, degenerative disease of the central nervous system (CNS) [1, 2] and the most common inflammatory neurological disease causing disability in early adulthood [3]. MS is characterized by relapses, disseminated lesions in the CNS, and a resulting progression of neurological disability that manifests in various neurological symptoms and signs. It substantially impacts the quality of life of those affected [4] and, in addition to the physical and psychological impairments, MS imposes high direct and indirect costs on the health care system [5]. In Germany, the prevalence was estimated at 337 per 100,000 population in 2019 [6]. Based on claims data, the age-adjusted (European Standard Population) incidence in 2012 was estimated to range from 6.6 to 21.8 per 100,000, depending on the case definition used [7]. In a comprehensive review, Lane et al. reported a wide range of incidence estimates worldwide with predominantly increasing MS incidence [8].

The causes of MS are not fully understood [9]. MS is deemed to be an autoimmune disease [10, 11], which is supported by the observation that the demyelination and subsequent degeneration of nerves within the CNS typical of MS is presumed to be an immune-mediated process, potentially caused by a viral infection [12]. Genetic predisposition, environmental and lifestyle factors, and their interaction constitute relevant risk factors in the development of MS [13]. According to a meta-estimation of hereditary and environmental factors on MS susceptibility, based on twin studies, heritability was estimated to account for 50% of the occurrence of MS. Shared environmental factors such as trans-generational epigenetic modifications or birth month accounted for 21%, and unshared environmental factors, e.g., infections, vitamin D (vit D) deficiency or smoking accounted for 29% of MS liability. The authors highlighted that the investigation of the influence of environmental factors as well as the respective individual lifestyle and infections, e.g., Epstein-Barr virus infection, should be the focus of future research [14]. In particular, early-life exposures that act on the immature immune system might be of relevance in the development of immune-mediated diseases such as MS [15]. The importance of early childhood factors has already been shown for type 1 diabetes [16], asthma [17] as well as for allergies [15, 18]. Of particular relevance in this context is the hygiene hypothesis, which states that individuals who have no or infrequent exposure to, e.g., infections that trigger an immune response, develop a less regulatory immune competence and are more susceptible to immune-mediated diseases [19]. The direct link between the total number of previous infections and the incidence of immune-mediated diseases is difficult to establish in observational studies since study participants remember previous infections only vaguely. Variables such as daycare attendance, number of siblings and birth order, contact with pets or livestock or growing up on a farm, and other social and economic indicators can be considered as surrogates instead [19]. However, these have provided conflicting results with regard to the occurrence of MS [20–26]. Other early childhood factors related to autoimmune diseases include prematurity, the mode of delivery, i.e., vaginal birth or cesarean section, and breastfeeding, all of which affect the development of the infant’s immune system [27, 28], but again studies on MS have yielded conflicting results regarding preterm birth [26, 29–33], mode of delivery [34–36] and breastfeeding [37]. As obesity leads to chronic low-grade inflammation [38], weight history during childhood and adolescence might be of relevance. Studies of weight history at the age of 10 [39] and 20 years [40–42], respectively, and also for birth weight [26, 30–32, 43, 44] have so far yielded conflicting results, especially when sex was taken into account. At last, stressful life events in childhood may play a role in the development of MS. Polick and colleagues conducted a systematic review, including twelve studies, most of which demonstrated an association between childhood trauma and subsequent MS. Physical and sexual abuse were the most common traumatic stressors reported in the included studies [45].

Since the causes of MS are not fully understood [9], and a large proportion of the risk might be explained by the interplay of modifiable risk factors [14], it is important to clarify the potential role of these factors. Hence, our study aimed to investigate the association between childhood and adolescence factors and MS risk.

## Methods

### Study sample

This work was based on data from the baseline assessment of the NAKO, a large population-based cohort study in Germany. The NAKO recruited approximately 205,000 individuals from 18 German study regions based on age and sex-stratified samples randomly drawn from the corresponding local registries of residents. As part of the standardized data collection, subjects underwent several biomedical examinations, participated in a face-to-face interview conducted by trained study assistants, and completed a self-administered touchscreen questionnaire [46]. A more detailed description of the design of the NAKO can be found elsewhere [46, 47]. Our analyses comprised participants who provided information on the presence of a physician-based MS diagnosis as well as on the covariates described below. Participants who answered "Don't know" or "No information" regarding an MS diagnosis were excluded. Furthermore, participants with MS were excluded if they did not provide information on age at MS diagnosis.

### Outcome ascertainment, exposure variables, and covariates

MS diagnosis and age at diagnosis were self-reported by NAKO participants in the rare diseases module of the face-to-face interview administered by trained interviewers.

Exposure variables and covariates were either collected during the face-to-face interview or by completion of a self-administered touchscreen questionnaire. The following two groups of exposures were considered with respect to the time of their occurrence – childhood and adolescence factors. Childhood factors include prematurity, born by cesarean section, birth weight, number of siblings, having had contact with pets and/or livestock during childhood, daycare attendance including age at first attendance, weight history reported as weight at the age of 10 years compared to peers as well as childhood trauma, measured by the Childhood Trauma Screener (CTS) [48]. The CTS is a 5-item brief childhood trauma assessment instrument developed from the original 28-item Childhood Trauma Questionnaire. The five items include the dimensions of emotional abuse, physical abuse, sexual abuse, emotional neglect, and physical neglect [48]. The summary score was used, ranging from 5 to 25 points, with higher scores indicating more severe trauma. Body Mass Index (BMI) at the age of 18 years—calculated from self-reported weight at that age and measured height at baseline—was included as an adolescence factor. We used the following thresholds: < 18.5 is equivalent to underweight, 18.5 to < 25 to normal weight, 25 to < 30 to overweight, and ≥ 30 kg/m^2^ to obesity. Data on childhood and adolescence exposures were collected as part of the self-administered questionnaire on touchscreen. Sex, education, migration status as well as birth year, categorized in ten-year birth cohorts (< 1955, 1955–1964, 1965–1974, 1975–1984, ≥ 1985), were considered as covariates in the analyses obtained in the face-to-face interview. Education was based on the International Standard Classification of Education 97 (ISCED) [49], with categories summarized as low (ISCED level 0–2), medium (ISCED level 3/4), and high (ISCED level 5/6) education.

### Statistical analyses

Descriptive analyses by MS status with respect to birth year, sex, education, migration background, age at onset of MS, and exposure variables were performed. Categorical variables were summarized as absolute and relative frequencies, and continuous variables were summarized as mean and standard deviation (SD). Missing values were displayed.

A multivariable Cox proportional hazards regression was performed to assess the association between childhood and adolescence factors and MS risk. Results are displayed as Hazard Ratios (HR) with corresponding 95% confidence intervals (CI). The outcome was defined as age at self-reported MS diagnosis. The observation period began at birth and ended either at the reported onset of MS in the case of an event or at age at the baseline examination date in the case of no event. The model was stratified by birth year to account for cohort effects, and adjusted for education and migration status. No violation of the proportional hazards assumption was detected by graphically examined Schoenfeld residuals.

The percentage of missing values across the predictor variables ranged from 0% to 43.2% in the group without MS and from 0% to 43.8% in the group with MS (see Table 1). In total, 75,247 records (36.8%) were complete. Multiple imputation was used to create and analyze 40 imputed datasets. Incomplete variables were imputed under fully conditional specification, using the default settings of the mice 3.0 package [50, 51]. HRs were estimated in each imputed dataset separately and subsequently pooled applying Rubin’s rules. We analyzed the data separately by sex to investigate sex-specific effects. For comparison, we also performed the analysis on the subset of complete cases.

**Table 1:** Characteristics of NAKO baseline participants and distribution of childhood and adolescence factors by MS status

**Variable**	**Persons without MS**^**a**^ **(*n* = 203415)**	**Persons with MS**^**a**^ **(*n* = 858)**
**Birth year**		
< 1955	45610 (22.4%)	125 (14.6%)
1955–1964	50907 (25.0%)	256 (29.8%)
1965–1974	55916 (27.5%)	278 (32.4%)
1975–1984	25551 (12.6%)	136 (15.9%)
≥ 1985	25431 (12.5%)	63 (7.3%)
**Sex**		
Female	102494 (50.4%)	579 (67.5%)
Male	100921 (49.6%)	279 (32.5%)
**Age at diagnosis (Mean (SD**^**a**^**))**	NA	36.3 (11.1)
**Education**		
Low	5107 (2.8%)	20 (2.6%)
Medium	146116 (79.0%)	606 (77.4%)
High	33716 (18.2%)	157 (20.1%)
Missing	18476	75
**Migration background**		
No	168796 (83.0%)	743 (86.6%)
Yes	34576 (17.0%)	115 (13.4%)
Missing	43	0
**Number of siblings**		
Only child	30926 (18.0%)	130 (18.5%)
1-2 sibling(s)	107814 (62.7%)	437 (62.3%)
≥ 3 siblings	33335 (19.4%)	134 (19.1%)
Missing	31340	157
**Premature birth (> 4 weeks before due date)**		
No	156024 (95.7%)	639 (95.5%)
Yes	7001 (4.3%)	30 (4.5%)
Missing	40390	189
**Cesarean section**		
No	155208 (94.4%)	629 (94.2%)
Yes	9292 (5.6%)	39 (5.8%)
Missing	38915	190
**Birth weight**		
Low	17531 (12.6%)	87 (14.8%)
Average	103066 (74.3%)	421 (71.6%)
High	18030 (13.0%)	80 (13.6%)
Missing	64788	270
**Ever breastfed**		
No	29536 (25.7%)	167 (34.6%)
Yes, ≤ 4 months	45024 (39.1%)	180 (37.3%)
Yes, > 4 months	40532 (35.2%)	135 (28.0%)
Missing	88323	376
**Contact with pets and/or livestock during childhood**		
No	87293 (50.8%)	348 (49.8%)
Yes	84668 (49.2%)	351 (50.2%)
Missing	31454	159
**Attended daycare**		
No	47393 (28.6%)	196 (29.2%)
Yes, 1st attendance at age 3-6 years	79977 (48.3%)	336 (50.1%)
Yes, 1st attendance at age 1- < 3 year(s)	28172 (17.0%)	98 (14.6%)
Yes, 1st attendance at age < 1 year	10143 (6.1%)	41 (6.1%)
Missing	37730	187
**Weight at the age of 10 years compared to peers**		
Lower	43328 (26.6%)	156 (23.5%)
Average	95681 (58.7%)	387 (58.2%)
Higher	24064 (14.8%)	122 (18.3%)
Missing	40342	193
**BMI**^**a**^** at the age of 18 years (kg/m**^**2**^**)**		
Underweight (< 18.5)	18283 (12.7%)	91 (15.7%)
Normal weight (18.5 - < 25)	108757 (75.8%)	398 (68.7%)
Overweight (25 - < 30)	13558 (9.4%)	68 (11.7%)
Obesity (≥ 30)	2884 (2.0%)	22 (3.8%)
Missing	59933	279
**Childhood Trauma**^**b**^** (Mean (SD))**	7 (2.7)	7 (3.0)
Missing	32174	161

^a^*MS* Multiple Sclerosis, *SD* Standard deviation, *BMI* Body Mass Index

^b^Assessed with the Childhood Trauma Screener (5 – 25 points)

All statistical analyses were performed with R version 4.2.0 (2022–04-22 ucrt) [52] using the packages “survival” [53], “survminer” [54], “sjPlot” [55], “MASS” [56], “mice” [50] and “micemd” [57].

## Results

Of the initial 204,899 NAKO participants, a total of 626 (0.3%) individuals were excluded because they either did not provide information on MS diagnosis (*n* = 624) or age at diagnosis (*n* = 2). Accordingly, a total of 204,273 subjects were included in the analysis – 858 (579 females, 279 males) with and 203,415 (102,494 females, 100,921 males) without an MS diagnosis.

Table 1 summarizes the characteristics as well as the distribution of childhood and adolescence factors separately for persons with and without MS.

Table 2 summarizes the results of the multivariable Cox regression.

**Table 2:** Multivariable Cox proportional hazards regression on the association between childhood and adolescence factors and multiple sclerosis

**Variable**	**Multivariable Cox proportional hazards model**^**a**^	
	**HR**^**b**^	**95% CI**^**b**^
**Sex**		
Female	*Ref*	
Male	0.48	0.41 – 0.56
**Number of siblings**		
Only child	*Ref*	
1-2 sibling(s)	0.93	0.73 – 1.17
≥ 3 siblings	0.81	0.61 – 1.09
**Premature birth (> 4 weeks before due date)**		
No	*Ref*	
Yes	0.94	0.61 – 1.47
**Cesarean section**		
No	*Ref*	
Yes	1.10	0.80 – 1.52
**Birth weight**		
Low	0.99	0.74 – 1.33
Average	*Ref*	
High	1.14	0.90 – 1.45
**Ever breastfed**		
No	*Ref*	
Yes, ≤ 4 months	1.02	0.79 – 1.31
Yes, > 4 months	0.88	0.69 – 1.11
**Contact with pets and/or livestock during childhood**		
No	*Ref*	
Yes	1.04	0.69 – 1.58
**Attended daycare**		
No	*Ref*	
Yes, 1st attendance at age 3-6 years	0.93	0.77 – 1.12
Yes, 1st attendance at age 1- < 3 year(s)	0.77	0.58 – 1.03
Yes, 1st attendance at age < 1 year	0.97	0.63 – 1.50
**Weight at the age of 10 years compared to peers**		
Lower	0.86	0.70 – 1.05
Average	*Ref*	
Higher	0.93	0.73 – 1.19
**BMI**^**b**^** at the age of 18 years (kg/m**^**2**^**)**		
Underweight (< 18.5)	1.12	0.89 – 1.42
Normal weight (18.5 - < 25)	*Ref*	
Overweight (25 - < 30)	2.03	1.41 – 2.94
Obesity (≥ 30)	1.89	1.02 – 3.48
**Childhood Trauma**^**c**^** (per 5 units)**	1.08	0.95 – 1.24

^a^Adjusted for education and migration status, stratified by birth year (categorized as: < 1955, 1955–1964, 1965–1974, 1975–1984, ≥ 1985)

^b^*HR* Hazard Ratio, *CI* Confidence Interval, *BMI* Body Mass Index

^c^Assessed with the Childhood Trauma Screener (5 – 25 points)

We observed a reduced risk of MS for men compared to women (HR 0.48, 95% CI 0.41 to 0.56).

Compared to normal weight at the age of 18 years, overweight (HR 2.03, 95% CI 1.41 to 2.94) and obesity (HR 1.89, 95% CI 1.02 to 3.48) at the age of 18 years were associated with a higher MS risk. No association with MS risk was observed for the remaining factors including number of siblings, prematurity, cesarean section, birth weight, breastfeeding, contact with pets and/or livestock, age at first daycare attendance, weight at the age of 10 years, and childhood trauma (Table 2).

In separate analyses by sex, we observed an association between overweight at the age of 18 years and an increased MS risk compared to normal weight at the age of 18 years in women (HR 1.55, 95% CI 1.05 to 2.29) but not in men. Furthermore, in contrast to no breastfeeding, breastfeeding duration of ≤ 4 months was related to a reduced risk of MS in men (HR 0.59, 95% CI 0.40 to 0.86) but not in women. Estimates for the remaining variables differed only slightly in women and men compared with the overall group (see Additional file 1).

When we restricted the analysis to complete cases (*n* = 75,247, 36.8% of the total cohort), we obtained similar results in both analyses of the total cohort and stratified by sex. However, due to the reduced sample size, the confidence intervals were considerably larger (see Additional files 2 & 3).

## Discussion

The present study was based on data from the baseline assessment of the population-based cohort study NAKO, which included 858 prevalent adult MS cases. We aimed to investigate associations between childhood and adolescence factors and MS risk. We observed associations between overweight and obesity at the age of 18 years and an increased risk of MS compared to normal weight at this age. In analyses stratified by sex, the association between overweight and increased MS risk remained for women but not for men. In contrast to no breastfeeding, a breastfeeding duration of ≤ 4 months was related to a reduced MS risk in men, but not in women. No association with MS risk was observed for the remaining childhood factors including number of siblings, prematurity, cesarean section, birth weight, contact with pets and/or livestock, age at first daycare attendance, weight at the age of 10 years, and childhood trauma.

Our results regarding the association of overweight and obesity with increased MS risk are in line with the meta-analysis of Liu et al. [41] and the study by Gianfrancesco and colleagues [40], both for the overall analysis and for the analyses stratified by sex. Contrary to what might be expected from our results, but in line with previous studies [26, 30–32, 43], we did not observe an association between MS risk and higher birth weight or higher weight at the age of 10 years compared with average weight. Thus, the time of onset of overweight or obesity may have an impact on MS risk. A possible explanation might be the interaction of weight and physical activity throughout childhood. Physical activity contributes to the prevention of overweight and obesity and the resulting anti-inflammatory effect can possibly prevent the development of inflammatory diseases in general [58]. However, physical activity often decreases during puberty [59]. While children still benefit from the positive effects of physical activity in regulating body weight, this may no longer be the case for adolescents due to lower levels of physical activity. In contrast, in a study based on retrospectively collected data of 1,944 persons with MS and 435,959 persons without MS from the UK Biobank sample, Belbasis and colleagues showed that "plumper than average body size" at the age of 10 years was associated with an increased risk of MS (Odds Ratio (OR) 1.38, 95% CI 1.21 to 1.58). The authors confirmed this result in a subsequent Mendelian Randomization study (OR 1.22, 95% CI 1.05 to 1.41) [39]. This clearly demonstrates the need for future research on the influence of the interaction between body weight and physical activity in childhood and adolescence on the risk of MS. Amidst the hygiene hypothesis [19], we would have expected to observe associations with factors such as a higher number of siblings, younger age at first daycare attendance, contact with pets and/or livestock and decreased MS risk as was reported for other autoimmune disorders [17] and allergies [15, 18]. Regarding the number of siblings, our results are consistent with those of Bager et al. [22] and Banwell et al. [23]. In contrast, one case–control study comprising 245 MS cases and 296 population-based controls reported a decreased risk of MS in participants with ≥ 3 older siblings, but only four participants with MS fell into this category in their study [20]. Another study found that an increasing duration of exposure to a younger sibling aged < 2 years in the first six years of life reduced MS risk [24]. These conflicting results highlight the continuing need for research into MS risk factors. We did not observe an association between daycare attendance and MS risk. In the study by Conradi and colleagues, a protective effect of daycare attendance at the age of 0–3 years on subsequent MS risk was found (OR 0.50, 95% CI 0.31 to 0.80) [20]. Contact with pets and/or farm animals, which has also been addressed in the context of the hygiene hypothesis as a potential factor influencing MS risk [19], was also not related to disease risk in our study, corroborating findings from a systematic review, which investigated the association between pet ownership during childhood and subsequent MS risk [25]. A meta-analysis on pet ownership in infancy and the incidence of the autoimmune diseases asthma and allergic rhinitis at school age also observed no association [60].

Our estimate regarding breastfeeding has a wide confidence interval, however, it is in line with our recent meta-analysis on the association between having been breastfed and MS risk (OR 0.86, 95% CI 0.75 to 0.99) [37]. In our meta-analysis, it also became apparent that the effect of breastfeeding on MS risk might differ between men and women, as shown in the included study by Hedström and colleagues, who observed that breastfeeding duration of ≥ 4 months compared to < 4 months was related to a reduced MS risk in men but not in women (OR 0.5, 95% CI 0.4 to 0.7) [61]. In our present analyses, we observed a HR of 0.59 (95% CI 0.40 to 0.86) for a breastfeeding duration of ≤ 4 months and a HR of 0.70 (95% CI 0.48 to 1.03) for a breastfeeding duration of > 4 months compared to no breastfeeding for men, suggesting that having been breastfed at all is the most relevant factor.

For the remaining childhood factors, i.e., prematurity, born by cesarean section, and childhood trauma no association with MS risk was observed. Regarding the first two factors, our results are consistent with the systematic review and meta-analysis by Badihian and colleagues, in which studies were summarized narratively for preterm birth and meta-analytically for the factor cesarean section, the latter yielding a pooled OR of 0.90 (95% CI 0.52 to 1.56) [34]. Regarding childhood trauma, our results are in contrast to the systematic review by Polick and colleagues [45]. However, the comparability of our results is limited by the fact that we only investigated the sum score of the CTS, but not the individual scales (emotional, physical, and sexual abuse and emotional and physical neglect scales) that comprise the sum score. Such an analysis of MS risk using the subscales of the CTS was beyond the scope of our study and will be incorporated as a main focus in subsequent work.

The primary strengths of this study comprise the large number of participants, its population basis, a comprehensive examination and assessment program that is performed by trained study personnel based on written standard operating procedures as well as the stringent quality assurance of the data collection and usage [46, 47]. The cohort enables analyses with both a sufficiently large group of individuals with MS and a wide range of potential risk factors and covariates to be considered, hence enabling us to contribute to the existing evidence regarding the associations between childhood and adolescence factors and the risk of MS.

Nevertheless, our study also has limitations. It cannot be excluded that the MS status as well as disease severity are related to participation readiness. Participants had to visit the respective study center for the examinations and the interview, therefore a selection of less severely affected MS cases might have occurred. Furthermore, severely affected individuals might have a different risk profile than less severely affected individuals. Hence, selection bias toward less severely affected cases may have resulted in an underestimation of associations in population-based MS cases as a whole. On the other hand, participants with MS and especially more severely affected individuals might be more motivated to participate in a study that investigates disease-related risk factors. Accordingly, future studies should account for the potential disease severity during the course of the disease. Also, as in all population-based cohort studies, there might be a healthy participant bias, implying that participants have a different risk factor pattern than the general population.

Moreover, the analyses are based on self-reported MS diagnoses. To our knowledge, no study to date has examined the sensitivity and specificity of self-reported MS diagnoses and reported HRs might be attenuated toward null. A validation study of the diagnoses, by use of information on treatment, medical records, and/or health insurance data is underway, however not been completed yet. Claflin and colleagues evaluated the consistency and validity of self-reported year of MS diagnosis among 2,245 participants in the Australian Multiple Sclerosis Longitudinal Study. 88% to 92% of respondents were able to recall their year of diagnosis with a deviation of ≤ 1 year. Thus, patient-reported year of diagnosis appears to be reliable information to use in analyses [62].

As early childhood factors were the main focus of our study, which for most study participants occurred on average 40 to 50 years ago, recall bias may have occurred. However, we assume that this is not a differential recall (thus, the bias not being different between MS cases and participants without MS), since the purpose of the data collection in the NAKO did not focus on MS and risk factors specific to MS at the time of recruitment, but rather on the investigation of widespread diseases such as cancer, diabetes mellitus, or cardiovascular diseases (see [47]). The observation that people with MS suffer increasingly from cognitive and memory dysfunction as the disease progresses could be more decisive than the pure temporal component with regard to a recall bias. However, due to the design of the NAKO, only individuals with a certain level of physical and cognitive fitness participated in the study. Therefore, we assume that the risk of a recall bias caused by this is rather low. However, if differential recall had occurred between participants with MS and without MS, the resulting bias was not strong, as our results are largely consistent with prospective studies of incident MS cases which have evaluated childhood and adolescence factors [30, 63, 64]. To some extent, our study might be biased due to a certain degree of misclassification (e.g., breastfeeding duration). Furthermore, some subgroups comprised only a few MS cases, thus the power here is low.

At the time of our analyses, it was not yet possible to determine the number of childhood-acquired infections by determining the viral load in the biosamples collected during the NAKO baseline examination. For this reason, we had to use surrogate variables such as number of siblings, daycare attendance, or pet ownership. As the determination of viral load via biospecimen analyses will be possible in the near future, the investigation of the interplay between viral load with childhood and adolescence factors is worth considering. Genetic factors may also be considered in these analyses.

Furthermore, we were unable to include other, lesser-known but potentially equally important risk factors such as month of birth, maternal vit D serum levels during pregnancy, or exposure to vaccinations in our analysis. For example, the month of birth, which is linked to the mother's exposure to sunlight during pregnancy and thus directly to the maternal vit D serum level, could be regarded as a precursor to the vit D level of the MS case. The link between vit D deficiency and an increased risk of MS has already been shown [65]. Thus, future studies should take these factors into account.

As shown in Table 1, many variables included in the analyses had a high proportion of missing values (e.g., “Ever breastfed” with 43.8% and 43.2% in persons with and without MS, respectively). It is difficult to clearly classify the type of missingness. However, following the classification by Rubin [66] missing values for most variables seem to be classifiable as missing completely at random. Hence, we used multiple imputation and compared the results with those of the complete case analysis. Both methods showed similar results, supporting the validity of the results using multiple imputation (see Additional file 2 & 3).

## Conclusions

In summary, based on this study the proportion of MS cases that can be explained by childhood and adolescence factors considered in this study was low. Nevertheless, we emphasize the observed association between overweight and obesity at the age of 18 years compared with normal weight at the age of 18 years and increased risk of MS. Stratified by sex, the association between overweight and increased MS risk was only found in women but not in men. Furthermore, a breastfeeding duration of ≤ 4 months compared to no breastfeeding was related to a reduced MS risk only in men. Given the large sample size, our study contributed to the existing evidence from previous studies. Our finding of an association between overweight and obesity during adolescence offers potential for MS prevention. In order to reduce the incidence of MS or at least delay its onset, the association between weight gain, onset of overweight and obesity in childhood and adolescence and their interaction with physical activity level should be investigated in longitudinal studies. In particular, sex-specific effects should be taken into account in future studies. Furthermore, the collection of biosamples during the NAKO baseline examination allows the investigation of the interplay of genetic factors, viral load, and childhood and adolescence factors in the future.

### Supplementary Information

**Additional file 1: Supplementary Table S1.** Multivariable Cox proportional hazards regression on the association between childhood and adolescence factors and multiple sclerosis stratified by sex (n_women_= 103073; n_men_ = 101200).

**Additional file 2: Supplementary Table S2.** Multivariable Cox proportional hazards regression on the association between childhood and adolescence factors and multiple sclerosis – complete case analysis for the total sample and stratified by sex.

**Additional file 3: Supplementary Figure S3.** Forest Plot: Multivariable Cox proportional hazards regression on the association between childhood and adolescence factors and multiple sclerosis – Imputation model and complete case analysis.

## Data Availability

The data that support the findings of this study are available from the NAKO e.V. but restrictions apply to the availability of these data, which were used under license for the current study, and so are not publicly available. Data are however available from the authors upon reasonable request and with permission of the NAKO e.V.

## Abbreviations

BMBF: German Federal Ministry of Education and Research
BMI: Body Mass Index
CI: Confidence Interval
CNS: Central nervous system
CTS: Childhood Trauma Screener
HR: Hazard Ratio
ISCED: International Standard Classification of Education
MS: Multiple Sclerosis
NAKO: German National Cohort
OR: Odds Ratio
SD: Standard deviation
Vit D: Vitamin D
