# Supplementary material for: Childhood and adolescence factors and multiple sclerosis: results from the German National Cohort (NAKO)
Source: BMC Neurol. 2024 Apr 13;24:123. doi: 10.1186/s12883-024-03620-4 (PMC11015562; doi:10.1186/s12883-024-03620-4)
Supplement: Supplementary file 1 — Additional file 1: Supplementary Table S1. Multivariable Cox proportional hazards regression on the association between childhood and adolescence factors and multiple sclerosis stratified by sex (nwomen= 103073; nmen = 101200). [file 12883_2024_3620_MOESM1_ESM.pdf]

Supplementary Table S1: Multivariable Cox proportional hazards regression on the association between childhood and adolescence factors and multiple sclerosis stratified by sex (n_{women} = 103073; n_{men} = 101200)

	**Multivariable Cox proportional Hazards Model[a]**			
	**Women**		**Men**	
**Variable**	**HR[b]**	**95% CI[b]**	**HR[b]**	**95% CI[b]**
**Number of siblings**				
Only child	*Ref.*		*Ref.*	
1-2 sibling(s)	0.94	0.75 – 1.19	1.03	0.72 – 1.47
≥3 siblings	1.00	0.74 – 1.34	0.87	0.55 – 1.37
**Premature birth (>4 weeks before due date)**				
No	*Ref.*		*Ref.*	
Yes	0.86	0.49 – 1.51	1.12	0.55 – 2.27
**Cesarean section**				
No	*Ref.*		*Ref.*	
Yes	1.07	0.70 – 1.65	1.14	0.67 – 1.92
**Birth weight**				
Low	1.04	0.72 – 1.48	1.08	0.60 – 1.93
Average	*Ref.*		*Ref.*	
High	1.02	0.72 – 1.44	1.28	0.89 – 1.83
**Ever breastfed**				
No	*Ref.*		*Ref.*	
Yes, ≤4 months	1.21	0.96 – 1.51	0.59	0.40 – 0.86
Yes, >4 months	0.85	0.65 – 1.09	0.70	0.48 – 1.03
**Contact with pets and/or livestock during childhood**				
No	*Ref.*		*Ref.*	
Yes	1.00	0.70 – 1.41	1.23	0.79 – 1.93
**Attended daycare**				
No	*Ref.*		*Ref.*	
Yes, 1st attendance at age 3-6 years	0.81	0.64 – 1.03	1.17	0.84 – 1.64
Yes, 1st attendance at age 1-<3 year(s)	0.78	0.57 – 1.07	1.00	0.64 – 1.57
Yes, 1st attendance at age <1 year	0.77	0.52 – 1.14	0.49	0.19 – 1.25
**Weight at the age of 10 years compared to peers**				
Lower	0.85	0.67 – 1.08	1.00	0.69 – 1.43
Average	*Ref.*		*Ref.*	
Higher	0.94	0.70 – 1.25	1.31	0.88 – 1.96
**BMI[b] at the age of 18 years (kg/m²)**				
Underweight (<18.5)	0.96	0.72 – 1.27	1.06	0.62 – 1.81
Normal weight (18.5 - <25)	*Ref.*		*Ref.*	
Overweight (25 - <30)	1.55	1.05 – 2.29	0.78	0.45 – 1.37
Obesity (≥30)	1.78	0.96 – 3.30	1.45	0.66 – 3.17
**Childhood Trauma[c] (per 5 units)**	1.11	0.96 – 1.29	1.05	0.81 – 1.37

[a]Adjusted for education and migration status, stratified by birth year (categorized as: <1955, 1955-1964, 1965-1974, 1975-1984, ≥1985)

[b]HR = Hazard Ratio, CI = Confidence Interval, BMI = Body Mass Index

[c]Assessed with the Childhood Trauma Screener (5 – 25 points)
