# Supplementary material for: Childhood and adolescence factors and multiple sclerosis: results from the German National Cohort (NAKO)
Source: BMC Neurol. 2024 Apr 13;24:123. doi: 10.1186/s12883-024-03620-4 (PMC11015562; doi:10.1186/s12883-024-03620-4)
Supplement: Supplementary file 2 — Additional file 2: Supplementary Table S2. Multivariable Cox proportional hazards regression on the association between childhood and adolescence factors and multiple sclerosis – complete case analysis for the total sample and stratified by sex. [file 12883_2024_3620_MOESM2_ESM.pdf]

Supplementary Table S2: Multivariable Cox proportional hazards regression on the association between childhood and adolescence factors and multiple sclerosis – complete case analysis for the total sample and stratified by sex

	**Multivariable Cox proportional Hazards Model[a]** (n = 75247; 310 participants with and 74937 participants without an MS[b] diagnosis)		**Multivariable Cox proportional Hazards Model – Women[a]** (n = 41296; 228 women with and 41068 women without an MS[b] diagnosis)		**Multivariable Cox proportional Hazards Model – Men[a]** (n = 33951, 82 men with and 33869 men without an MS[b] diagnosis)	
**Variable**	**HR[b]**	**95% CI[b]**	**HR[b]**	**95% CI[b]**	**HR[b]**	**95% CI[b]**
**Sex**						
Female	*Ref.*					
Male	0.43	0.33 – 0.56				
**Number of siblings**						
Only child	*Ref.*		*Ref.*		*Ref.*	
1-2 sibling(s)	1.16	0.85 – 1.58	1.07	0.75 – 1.53	1.47	0.75 – 2.89
≥3 siblings	1.05	0.70 – 1.57	1.10	0.69 – 1.73	0.94	0.39 – 2.27
**Premature birth (>4 weeks before due date)**						
No	*Ref.*		*Ref.*		*Ref.*	
Yes	1.10	0.62 – 1.96	0.95	0.48 – 1.86	1.83	0.58 – 5.80
**Cesarean section**						
No	*Ref.*		*Ref.*		*Ref.*	
Yes	1.23	0.77 – 1.97	1.27	0.73 – 2.21	1.14	0.48 – 2.68
**Birth weight**						
Low	1.15	0.80 – 1.65	1.24	0.84 – 1.83	0.78	0.29 – 2.12
Average	*Ref.*		*Ref.*		*Ref.*	
High	1.06	0.75 – 1.49	1.09	0.72 – 1.67	0.99	0.56 – 1.75
**Ever breastfed**						
No	*Ref.*		*Ref.*		*Ref.*	
Yes, ≤4 months	1.08	0.82 – 1.43	1.25	0.92 – 1.69	0.71	0.38 – 1.30
Yes, >4 months	0.99	0.73 – 1.34	0.90	0.63 – 1.30	1.06	0.58 – 1.91
**Contact with pets and/or livestock during childhood**						
No	*Ref.*		*Ref.*		*Ref.*	
Yes	1.06	0.85 – 1.33	1.00	0.77 – 1.30	1.30	0.84 – 2.02
**Attended daycare**						
No	*Ref.*		*Ref.*		*Ref.*	
Yes, 1st attendance at age 3-6 years	0.91	0.68 – 1.22	0.84	0.60 – 1.19	1.13	0.63 – 2.03
Yes, 1st attendance at age 1-<3 year(s)	0.84	0.57 – 1.25	0.89	0.56 – 1.41	0.78	0.36 – 1.68
Yes, 1st attendance at age <1 year	0.85	0.52 – 1.38	0.97	0.58 – 1.65	0.36	0.08 – 1.58
**Weight at the age of 10 years compared to peers**						
Lower	0.89	0.67 – 1.18	0.80	0.56 – 1.13	1.13	0.67 – 1.92
Average	*Ref.*		*Ref.*		*Ref.*	
Higher	1.01	0.73 – 1.40	0.95	0.65 – 1.38	1.18	0.62 – 2.25
**BMI[b] at the age of 18 years (kg/m^2)**						
Underweight (<18.5)	1.23	0.89 – 1.70	1.16	0.81 – 1.66	2.09	1.01 – 4.33
Normal weight (18.5 - <25)	*Ref.*		*Ref.*		*Ref.*	
Overweight (25 - <30)	1.93	1.35 – 2.75	2.37	1.57 – 3.58	1.21	0.60 – 2.45
Obesity (≥30)	3.02	1.70 – 5.39	2.91	1.42 – 5.93	3.41	1.26 – 9.24
**Childhood Trauma[c] (per 5 units)**	0.94	0.75 – 1.18	0.91	0.70 – 1.18	1.05	0.65 – 1.70

[a]Adjusted for education and migration status, stratified by birth year (categorized as: <1955, 1955-1964, 1965-1974, 1975-1984, ≥1985)

[b]MS = Multiple Sclerosis, HR = Hazard Ratio, CI = Confidence Interval, BMI = Body Mass Index

[c]Assessed with the Childhood Trauma Screener (5 – 25 points)
