# Supplementary material for: Childhood and adolescence factors and multiple sclerosis: results from the German National Cohort (NAKO)
Source: BMC Neurol. 2024 Apr 13;24:123. doi: 10.1186/s12883-024-03620-4 (PMC11015562; doi:10.1186/s12883-024-03620-4)

Supplementary Figure S3: Forest Plot: Multivariable Cox proportional hazards regression on the association between childhood and adolescence factors and multiple sclerosis – Imputation model and complete case analysis

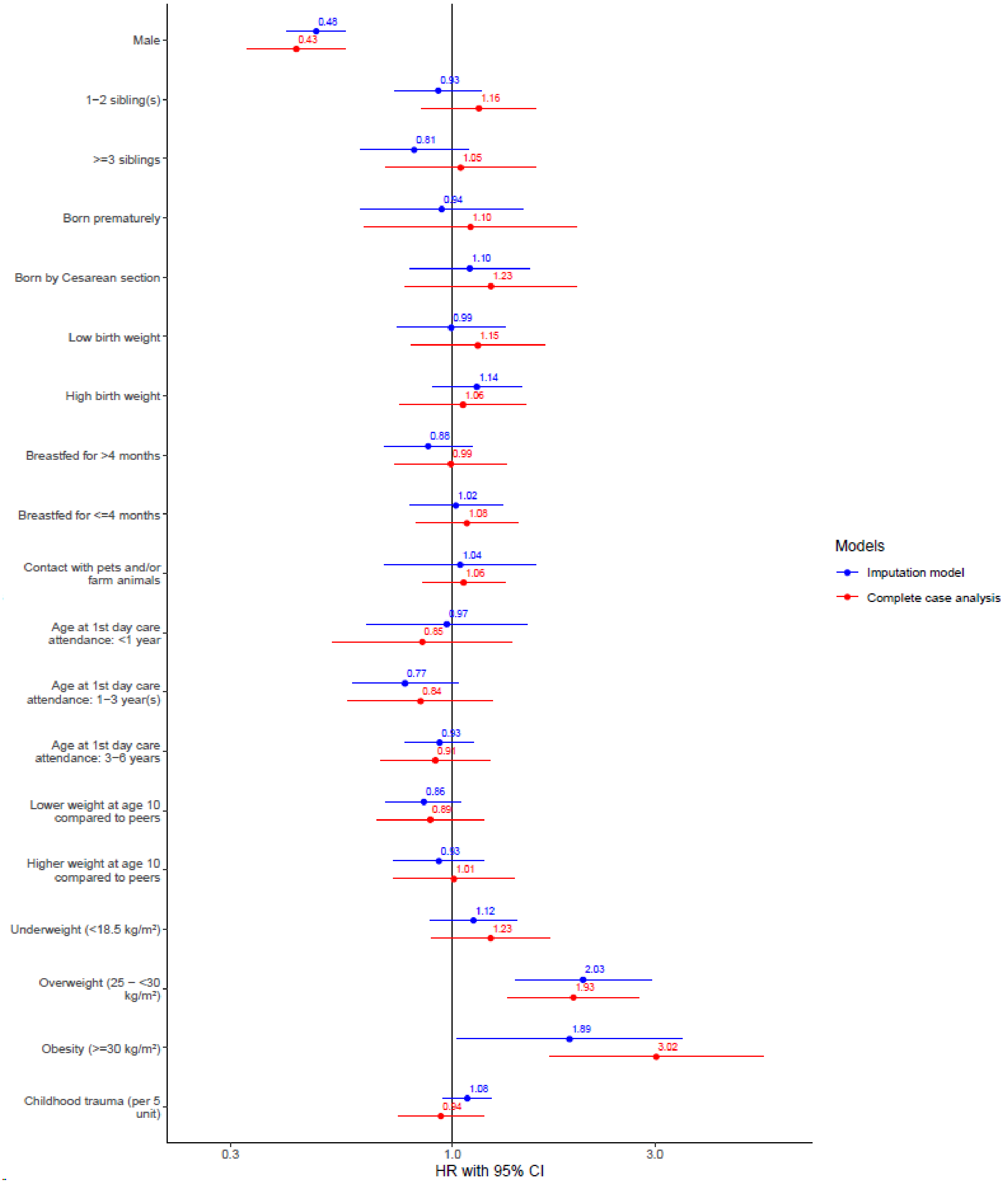

Supplement: Supplementary file 3 — Additional file 3: Supplementary Figure S3. Forest Plot: Multivariable Cox proportional hazards regression on the association between childhood and adolescence factors and multiple sclerosis – Imputation model and complete case analysis. [file 12883_2024_3620_MOESM3_ESM.pdf]
